# Postnatal development of layer III pyramidal cells in the primary visual, inferior temporal, and prefrontal cortices of the marmoset

**DOI:** 10.3389/fncir.2013.00031

**Published:** 2013-03-08

**Authors:** Tomofumi Oga, Hirosato Aoi, Tetsuya Sasaki, Ichiro Fujita, Noritaka Ichinohe

**Affiliations:** ^1^Department of Ultrastructural Research, National Institute of Neuroscience, National Center of Neurology and PsychiatryKodaira, Tokyo, Japan; ^2^Laboratory for Cognitive Neuroscience, Graduate School of Frontier Biosciences, Osaka UniversityToyonaka, Osaka, Japan; ^3^Center for Information and Neural Networks, National Institute of Information and Communications Technology, Osaka UniversityYamadaoka, Suita, Osaka, Japan

**Keywords:** autism, area differences, dendritic spine, schizophrenia, spinogenesis

## Abstract

Abnormalities in the processes of the generation and/or pruning of dendritic spines have been implicated in several mental disorders including autism and schizophrenia. We have chosen to examine the common marmoset (*Callithrix jacchus*) as a primate model to explore the processes. As a first step, we studied the postnatal development of basal dendritic trees and spines of layer-III pyramidal cells in the primary visual sensory cortex (V1), a visual association cortex (inferior temporal area, TE), and a prefrontal cortex (area 12, PFC). Basal dendrites in all three areas were longer in adulthood compared with those in the newborn. In particular, rapid dendritic growth occurred in both TE and PFC around the second postnatal month. This early growth spurt resulted in much larger dendritic arbors in TE and PFC than in V1. The density of the spines along the dendrites peaked at 3 months of age and declined afterwards in all three areas: the degree of spine pruning being greater in V1 than in TE and PFC. The estimates of the total numbers of spines in the basal dendrites of a single pyramidal cell were larger in TE and PFC than in V1 throughout development and peaked around 3 months after birth in all three areas. These developmental profiles of spines and dendrites will help in determining assay points for the screening of molecules involved in spinogenesis and pruning in the marmoset cortex.

## Introduction

Investigating the mechanisms underlying neuropsychiatric disorders, including autism spectrum disorders, Rett syndrome, and schizophrenia is a rapidly progressing area in neuroscience. Recent studies have identified many genes and copy number variants related to these disorders, and these are suggested to cause morphological and functional synaptic abnormalities, circuit alterations, changes in excitatory and inhibitory balance, and behavioral symptoms (van Spronsen and Hoogenraad, 2010; Penzes et al., 2011; Levenga and Willemsen, 2012). Among these altered endophenotypes, an abnormal number of spines is an important pathogenesis shared by several neuropsychiatric disorders (Kaufmann and Moser, 2000; Penzes et al., 2011).

In the cerebral cortex of macaque monkeys and humans, the number of dendritic spines rapidly increases after birth and peaks around the end of the neonatal period or in an early phase of the infantile period (Huttenlocher, 1990; Petanjek et al., 2008, 2011; Elston et al., 2009, 2010a,b, 2011). Spine density then decreases during the later infantile period and adolescence period to reach the adult level, suggesting that the pruning of existing spines exceeds the generation of new spines in this period (Missler et al., 1993). This overshoot-type time course of spine formation and pruning has attracted the attention of researchers because of its possible involvement in developmental disorders and psychiatric diseases (Penzes et al., 2011). In autism, excessive spine formation and/or incomplete spine pruning may occur in childhood, which has been discussed as possibly providing a diagnostically useful increase in spine number (Hutsler and Zhang, 2010). In Rett syndrome and several other developmental disorders with autism-like symptoms, dendrites, and spines in pyramidal neurons decrease in number (Kaufmann and Moser, 2000). In subjects with schizophrenia, the over-pruning of spines during late childhood or adolescence has been implicated in the findings of a decreased number of spines (Glantz and Lewis, 2000; Kalus et al., 2000; Broadbelt et al., 2002; Garey, 2010). An understanding of the molecular underpinnings of spine pathology may provide insights into the etiology of these diseases and lead to the discovery of new drug targets.

The common marmoset (*Callithrix jacchus*) has become an increasingly important animal model in studies of the neuronal mechanisms of neuropsychiatric disorders involving social and emotional impairments. The reason is several-fold. First, there is the possibility of applying transgenic manipulations to this species (Sasaki et al., 2009). Second, this species rapidly matures, which makes developmental studies feasible. Third, the marmoset has rich social communication, which is critically devastated in autism spectrum disorders (Snowdon, 1997; Eliades and Wang, 2008; Agustín-Pavón et al., 2012). Finally, rodents do not show the peaking (overshoot-type) profile of dendritic spine development characteristic of the primate cerebral cortex (Micheva and Beaulieu, 1996), and they may not represent a model suitable for examining the relationship between spine development and neuropsychiatric disorders.

In this study, we studied the developmental profiles of the basal dendritic trees and spines of layer-III pyramidal cells in the primary visual sensory cortex (V1), a visual association cortex (inferior temporal area, TE), and an executive control area (area 12 of the prefrontal cortex, PFC) of the common marmoset. We found that all three areas showed an overshoot-type pattern of spine development, which was similar to that seen in humans and macaques. Basal dendrites in all three areas became longer after birth. In particular, neurons in TE and PFC exhibited dramatic growth of the basal dendrites during the first 2 months after birth; the basal dendrites of layer-III pyramidal cells in TE and PFC thus covered a wider cortical area than those in V1. The three areas exhibited similar developmental changes in the time courses of the density of spines along dendrites with a peak in the third month after birth. In addition, the estimated total number of spines on basal dendrites peaked at 3 months of age in all three areas, and it was nearly three times larger in TE and in PFC than in V1. The developmental time courses of the dendrites and spines and the areal differences will help to determine assay points for the future screening of the molecules that are involved in spinogenesis and pruning in the marmoset cortex.

## Materials and methods

As shown in Table 1, we used common marmosets (*Callithrix jacchus*) of either sex with the following ages: postnatal day 0 (0D; *n* = 1), 2 postnatal months (2M; *n* = 3), 3 postnatal months (3M; *n* = 1), 6 postnatal months (6M; *n* = 1), and 4.5 postnatal years (4.5Y; *n* = 1). All animal care procedures and experiments were conducted in accordance with protocols that were approved by the ethics committee for primate research at the National Center of Neurology and Psychiatry in Japan and that were in compliance with the National Institutes of Health Guide for the Care and Use of Laboratory Animals.

**Table 1:** **Vital data of the animals used in the present study**.

**Age**	**Animal**	**Sex**	**Body weight (g)**
0D	CJ4	Female	27.0
2Ma	CJ6	Female	96.4
2Mb	CJ9	Female	95.3
2Mc	CJ13	Male	94.2
3M	CJ3	Male	101.0
6M	CJ5	Male	194.2
4.5 Y	CJ1	Male	301.0

Note: 0D, 0 days of age; 2M, 2 months of age; 3M, 3 months of age; 6M, 6 months of age; and 4.5 years of age.

The above-mentioned time points were selected according to the following rationale. The 0D animal was examined because of its few visual experiences. McKinnell et al. (2001) have proposed that the life of the common marmoset can be divided into three periods based on testosterone blood concentrations, which are particularly important for androgen-related signaling. The three periods have been referred to as neonatal, infantile, and peripubertal (McKinnell et al., 2001). Thus, the 2M point is the time when plasma testosterone levels markedly decrease, which is a sign of the end of the neonatal period (McKinnell et al., 2001). The age of 3M corresponds to the time between the neonatal and infantile periods, and the age of 6M corresponds to the time between the infantile and prepubertal periods (McKinnell et al., 2001). Finally, four-and-a-half years (4.5 Years) corresponds to a mature adult (Chandolia et al., 2006).

**Figure 1:**
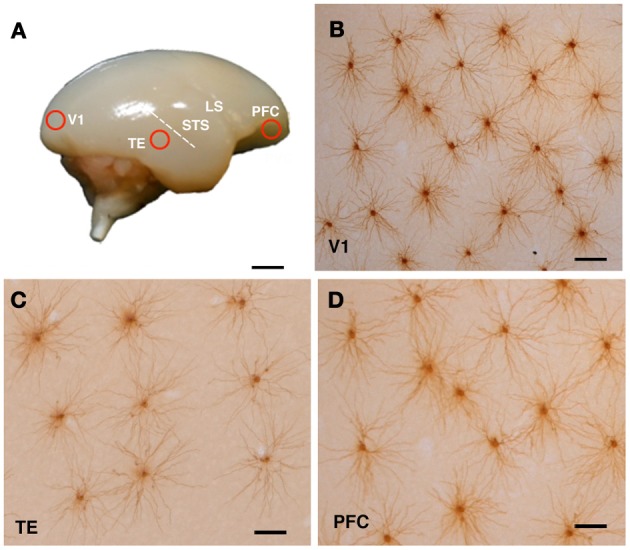
**Cortical sampling position (A) and labeled cells (B–D).** The animals were lightly fixed with paraformaldehyde, and the brains were removed, trimmed and flattened, and sliced at 250 μm tangential to the cortical surface. Slices were incubated in 4,6-diamidino-2-phenylindole solution to visualize neuronal nuclei. Layer III pyramidal cells were individually injected with Lucifer yellow and reacted for diaminobenzidine product **(B–D)**. Examples of injected cells from the primary visual cortex (V1), the inferior temporal cortex (TE), and the prefrontal cortex (PFC) of a 3-month-old monkey are shown in **(B)**, **(C)**, and **(D)**, respectively. Abbreviations: LS, lateral sulcus; STS, superior temporal sulcus. Scale Bars = 300 mm **(A)**, 100 μm **(B–D)**.

The animals were sedated with ketamine hydrochloride (Ketalar, 25 mg/kg i.m.; Daiichi Sankyo Co., Ltd., Tokyo, Japan) and overdosed with sodium pentobarbital (Nembutal, 75 mg/kg i.p.; Dainippon Sumitomo Pharma Co., Ltd., Osaka, Japan). The animals were perfused intracardially with 0.1 M potassium phosphate-buffered saline (pH 7.2), followed by 4% paraformaldehyde (Merck, Whitehouse Station, NY). Blocks of tissue were excised from the posterior part of the occipital operculum, which corresponded to the central 5–8° of the visual field representation in V1 (Fritsches and Rosa, 1996), the inferior temporal gyrus (TE) 2 mm ventral to the posterior edge of the superior temporal sulcus (Kaas, 1997), which corresponded to area ITd (Rosa et al., 2009) or TE3 (Paxinos et al., 2011), and the ventrolateral convexity of PFC, which corresponded to the area 12l + 12v (Burman and Rosa, 2009) or the area 12L + 12M (Paxinos et al., 2011) (Figure 1A). Note that area 12 in these references corresponds to area 10 of Brodmann (1909). The blocks were trimmed, flattened, and sliced parallel to the cortical surface at a thickness of 250 μm. Slices were incubated in 4,6-diamidino-2-phenylindole (DAPI; Sigma-Aldrich Co. LLC, St. Louis, MO) solution to visualize the cell bodies.

Pyramidal cells were individually injected with 8% Lucifer Yellow (Sigma-Aldrich Co. LLC, St. Louis, MO) under the visual guidance of ultraviolet illumination (Elston et al., 1997). All cell bodies of the injected cells were located in the supragranular layer, which was immediately above the granular layer and easily identified in 0.1% DAPI-stained preparations (Elston and Rosa, 1997). Only cells located in the lower part of layer III were injected.

The sections were processed with a biotinylated antibody (Life Technologies Corporation, Grand Island, NY) that was raised against Lucifer Yellow at a concentration of 1:5000 in a stock solution (2% bovine serum albumin, 1% Triton X-100, and 5% sucrose in 0.1 M phosphate buffer). They were then processed with a biotin-horseradish peroxidase complex (GE Healthcare Biosciences, Pittsburgh, PA; 1:100 in 0.1 M phosphate buffer) and reacted to obtain a light-stable reaction product of 3,3′-diaminobenzidine tetrahydrochloride (DAB; Sigma-Aldrich Co. LLC, St. Louis, MO). Figures 1B–D shows examples of injected cells in V1, TE, and PFC of a 3M monkey.

**Figure 2:**
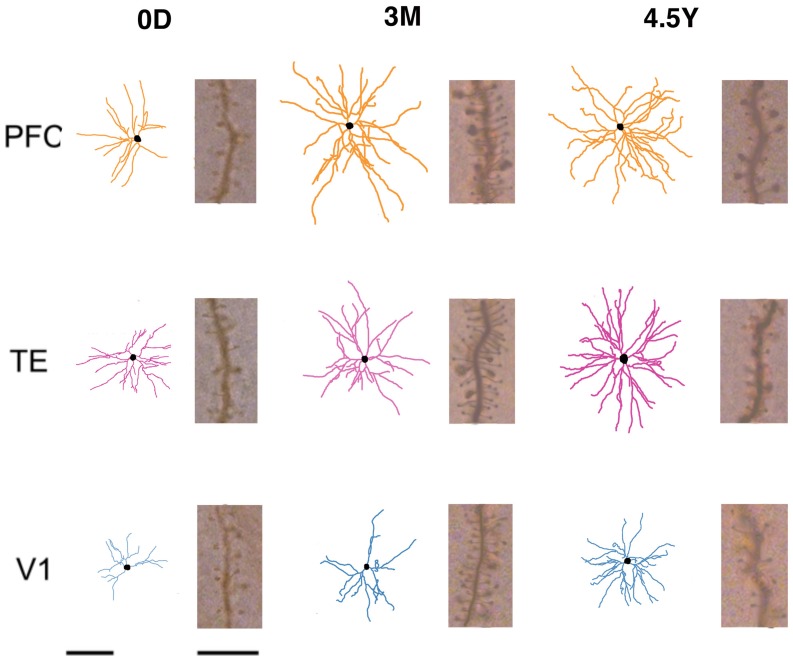
**Skeletonized, tangential, reconstructions of the basal dendritic trees of layer-III pyramidal cells (in the tangential plane) sampled from the primary visual (V1), inferior temporal visual association (TE), and prefrontal (PFC) cortices at day 0 (0D), 3 months (3M), and adulthood (4.5Y).** The illustrated cells represent the average of all cells sampled for each cortical area/age group. Insets illustrate the spine density for cells of each cortical area/age group at 50–70 μm from the cell body. Left Scale Bars = 100 μm (for all of skeletonized reconstructions of the basal dendritic trees of pyramidal cells), Right Scale Bars = 10 μm (for all of Insets illustrate the spine density for cells of each cortical area/age group at 50–70 μm from the cell body).

For the analysis, we included only those cells that had an unambiguous apical dendrite, had their complete basal dendritic arbors contained within the section, and were well filled. The basal dendritic trees of 324 cells from V1, TE, and PFC were reconstructed in two dimensions with a computer-aided tracing system (Neurolucida, MBF Bioscience, Williston, VT; Figure 2). Only the basal dendrites that could be verified as being issued from the cell body were included in the analyses. Two-dimensional reconstructions were selected for these analyses for the data to be compared directly with previous studies on macaque cerebral cortex (Elston et al., 2009, 2010a,b, 2011). Five to forty-seven cells were included in the analyses of each area/age group (Table 2). The branching structures of the dendritic trees were determined by Sholl analyses (Sholl, 1955).

**Table 2:** **Vital data of the animals used in the present study**.

Age	0D	2Ma	2Mb	2Mc	3M	6M	4.5Y
Animal	CJ4	CJ6	CJ8	CJ13	CJ3	CJ5	CJ1
V1	19	12	–	–	24	16	35
TE	14	5	5	6	17	34	47
PFC	6	6	6	–	22	15	32

Note: 0D, 0 days of age; 2M, 2 months of age; 3M, 3 months of age; 6M, 6 months of age; 4.5Y, 4.5 years of age.

Spine densities were calculated by drawing the horizontally projecting dendrites of randomly selected cells in their entirety while visualizing them with a Nikon 100X oil immersion objective (numerical aperture, 1.40) and then counting the number of spines per 10-μm segment (Eayrs and Goodhead, 1959; Valverde, 1967). Spine density was quantified as a function of distance from the cell body to the distal tips of the dendrites. We selected horizontally projecting dendrites for our calculations of spine densities to avoid trigonometric errors. Spine densities were calculated per 10-μm interval along the entire length of 20 randomly selected dendrites in each cortical area for each age group. No distinction was made between the different spine types (e.g., sessile or pedunculated spines).

The mean total number of spines within the basal dendritic trees of the cells in each cortical area/age was estimated by summing the products of the mean intersections of each 10 μm of Sholl annuli of the cells (see above) multiplied by the mean spine densities of each 10 μm of the corresponding distance from the cell body of 20 dendrites (for technical details, see Elston, 2001).

For tests of the size of the dendritic trees and somata, each cell was represented by a single data point. For each cell, Sholl and spine density analyses produced data of the number of intersections of Sholl annuli in each 10-μm interval from the cell body center as well as of the spine density in each 10-μm distance from the cell body center. Thus, we used repeated measures analysis of variance (ANOVA) tests for the statistical analyses of each area/age (Elston et al., 1997). For comparisons between two data groups, Student's *t*-tests were conducted, and we reported the *p* values. When other statistical methods (Mann–Whitney U-Test, One-Way ANOVA, repeated measures ANOVA) were performed, we specified them as such. These statistical tests were performed with StatView (SAS Institute, Inc., Cary, NC), and we described the results as *p* < 0.05, *p* < 0.001, or *p* < 0.0001 with other information (e.g., *F* value: *F*-test plays an important role in the ANOVA, and *F* value assesses whether any of the sampled groups is on average superior or inferior to the others vs. the null hypothesis that all groups yield the same mean; *U* value: Mann–Whitney's *U*-test is a non-parametric statistical test for assessing whether one of two samples of independent observations have larger values than the other. *U* value assesses how far the measured data are higher in one group than in the other.). We set the statistically significant level to *p* = 0.05.

## Results

A total of 324 pyramidal cells in layer III were included in the analyses (Table 2). The following results were based on 50,098 individual dendritic spines that were drawn and tallied.

### Basal dendritic field areas and total basal dendritic lengths

**Figure 3:**
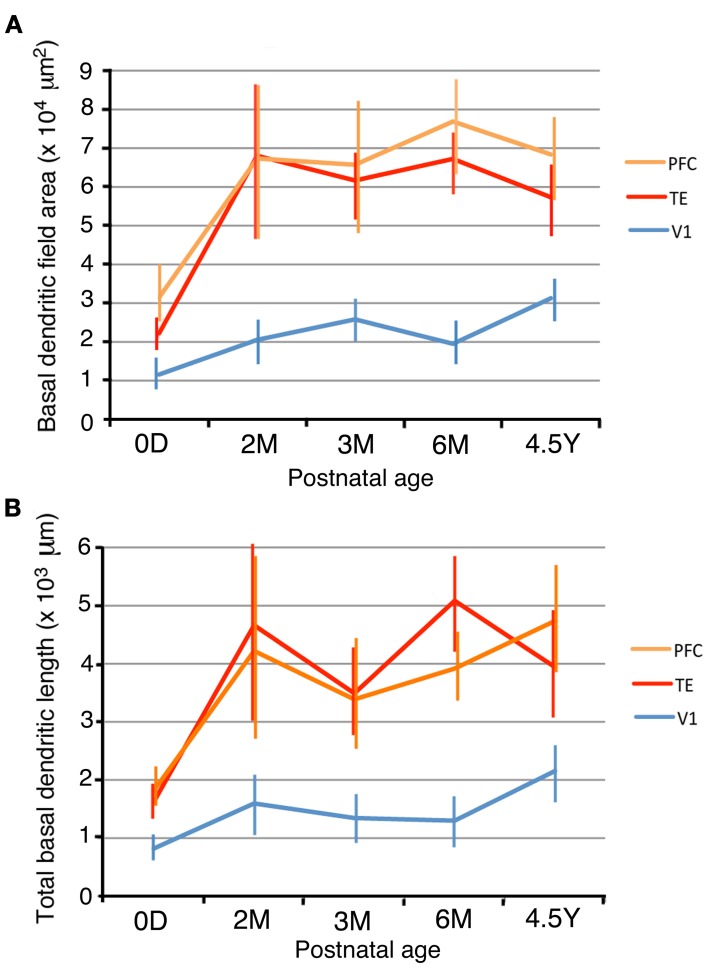
**Dendritic field areas (A) and total dendritic lengths (B) of layer-III pyramidal cells in V1, TE, and PFC at 0D, 2M, 3M, 6M, and 4.5Y.** Error bars indicate standard deviations.

In order to examine the development of dendritic arbors, we measured basal dendritic field areas and the entire dendritic lengths that were summed across the basal dendrites. V1, TE, and PFC differed in their growth profiles (Figure 2). The dendritic field areas of the pyramidal cells in V1 gradually increased from 0D to 4.5Y, except for a small decline from 3M to 6M (Figure 3A; One-Way ANOVA, *p* = 2.8 × 10^−18^). The dendritic field area at 4.5Y was 167% larger than that at 0D. In contrast, the dendritic field areas of TE and PFC showed a marked increase from 0D to 2M (TE: 319% increase, *p* = 4.3 × 10^−7^; PFC: 209% increase, *p* = 0.002) and then remained unchanged. Dendritic field areas of TE and PFC were larger at 4.5Y than at 0D (TE: 264% larger; *p* = 4.3 × 10^−7^; PFC: 200% larger, *p* = 2.9 × 10^−8^). Statistical analyses (One-Way ANOVAs) revealed that the differences in the sizes of the basal dendritic trees of pyramidal cells in any given cortical area were significant across the age groups (*p* < 0.001; V1, *F*_4_ = 28.00; TE, *F*_4_ = 33.15; PFC, *F*_4_ = 7.67).

Because of the different developmental profiles of the dendritic field areas between the three cortical areas and of the different sizes at 0D, there were relative differences in the dendritic field areas among the cortical areas at any given age (Figure 3A). For example, at 0D, the rank order of the dendritic field areas was PFC, TE, and V1. The averaged dendritic field area of PFC neurons was 302% larger than that in V1 (*p* = 4.9 × 10^−9^), and the averaged dendritic field area in TE was 195% larger than that in V1 (*p* = 1.4 × 10^−7^). Throughout the developmental course, the rank order did not change. At 4.5Y, the dendritic field areas of TE and PFC neurons were 269% and 324% larger than those of V1, respectively (*t*-test; *p* = 1.3 × 10^−24^ for TE vs. V1, *p* = 1.3 × 10^−24^ for PFC vs. V1). Statistical analyses (One-Way ANOVAs) revealed that the sizes of the dendritic trees of cells at any given age were significantly different among the cortical areas in all animals (*p* < 0.001; 0D, *F*_2_ = 7.32; 2M, *F*_2_ = 14.19; 3M, *F*_2_ = 60.72; 6M, *F*_2_ = 192.23; 4.5Y, *F*_2_ = 114.36).

The total basal dendritic lengths that were summed across the basal dendrites exhibited developmental changes similar to those of the basal dendritic field area (Figures 3A,B). The total dendritic lengths in TE and PFC markedly increased from 0D to 2M (*t*-test, *p* = 1.8 × 10^−7^ for TE and 0.014 for PFC). After 2M, the dendritic lengths in TE and PFC stayed almost at the same level until 4.5Y. In contrast, the dendritic lengths of V1 neurons gradually increased from 0D to 4.5Y. The dendrites in TE and PFC were markedly longer than those in V1 throughout development. One-Way ANOVAs revealed that the differences in the total lengths of the basal dendritic trees of pyramidal cells in any given cortical area were significant across the age groups (*p* < 0.001; V1, *F*_4_ = 37.81; TE, *F*_4_ = 32.73; PFC, *F*_4_ = 12.01). Similarly, One-Way ANOVAs revealed that the sizes of the dendritic trees of cells at any given age were significantly different among the cortical areas in all animals (*p* < 0.001; 0D, *F*_2_ = 34.31; 2M, *F*_2_ = 17.46; 3M, *F*_2_ = 49.46; 6M, *F*_2_ = 137.80; 4.5Y, *F*_2_ = 93.49).

### Branching patterns of the basal dendritic trees

**Figure 4:**
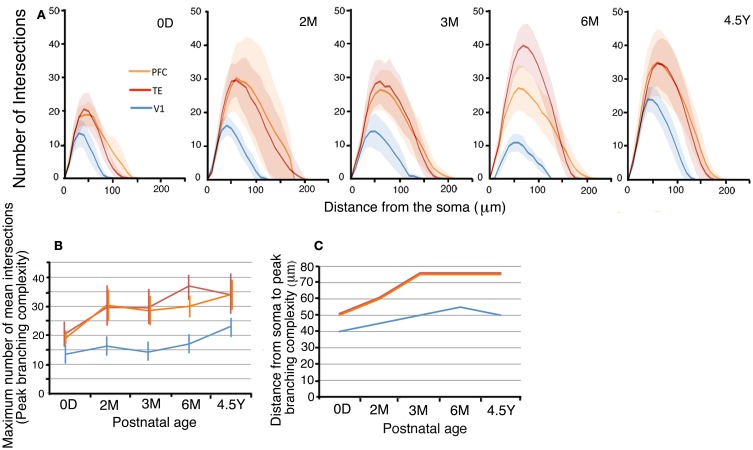
**(A)** Sholl plots of the branching patterns of the basal dendritic trees of layer-III pyramidal cells from V1, TE, and PFC. The shaded areas indicate the standard deviations. **(B,C)** Developmental changes in the peak number of Sholl intersections, and the distance from the soma to the position of the peak number of Sholl intersections of layer-III pyramidal cells. Error bars indicate standard deviations.

The branching complexities, as evidenced by their Sholl profiles, changed with age in all three areas (Figures 4A,B). We first examined the maximum number of mean dendritic intersections with Sholl annuli (peak branching complexity; Figure 4B). In V1, the peak branching complexity increased from 0D to 2M, then decreased to 6M, and finally exhibited the largest value at 4.5Y. The peak branching complexity of neurons in the PFC paralleled the changes in V1. TE exhibited the highest complexity at 6M. At 4.5Y, the peak branching complexity in TE and PFC showed a comparable value. Throughout development, V1 neurons showed a substantially lower complexity than TE and PFC neurons. At 0D, the peak numbers of dendritic intersections with Sholl annuli were 146% and 152% larger in TE and PFC, respectively, than in V1. At 4.5Y, the numbers were 136% and 138% larger in TE and PFC, respectively, than in V1. Comparisons of the branching complexities of the dendritic trees of pyramidal cells among the cortical areas at each given age revealed that cells in TE and PFC had higher numbers of branches than those in V1 at all corresponding ages (*p* < 0.001). Except at 0D between PFC and V1, complexity was significantly different between TE and V1 and between PFC and V1 at any ages (Figure 4A; Mann–Whitney *U-test*: 0D, TE vs. V1, *U* = 97,886, *n*_1_ = 378, *n*_2_ = 475; 2M, TE vs. V1, *U* = 165,049, *n*_1_ = 1340, *n*_2_ = 300, PFC vs. V1; *U* = 14,651, *n*_1_ = 182, *n*_2_ = 300; 3M, TE vs. V1, *U* = 163,875, *n*_1_ = 680, *n*_2_ = 744, PFC vs. V1, *U* = 249,610, *n*_1_ = 968, *n*_2_ = 744; 6M, *U* = 156,418, *n*_1_ = 1360, *n*_2_ = 384, PFC vs. V1, *U* = 105,281, *n*_1_ = 720, *n*_2_ = 384; 4.5Y, TE vs. V1, *U* = 541,615, *n*_2_ = 1598, *n*_2_ = 980, PFC vs. V1, *U* = 100,920, *n*_2_ = 576, *n*_2_ = 980).

In addition, the distance from the soma to the Sholl annulus with the peak branching complexity increased with age (Figure 4C). In V1, the distance of the peak dendritic complexity from the soma gradually increased from 0D to 6M and then remained nearly the same until 4.5Y. In TE and PFC, the distance increased from 0D to 3M and then remained at the same level from 3M to 4.5Y.

Lastly, the statistical analysis (One-Way repeated measures ANOVA) revealed that the numbers of dendritic intersections with Sholl annuli were significantly different among the cortical areas (*p* < 0.001) at each given age (0D, *F*_2_ = 39.58; 2M, *F*_2_ = 16.67; 3M, *F*_2_ = 54.85; 6M, *F*_2_ = 136.32; 4.5Y, *F*_2_ = 92.99: Figure 4A).

### Spine densities and numbers on basal dendrites

**Figure 5:**
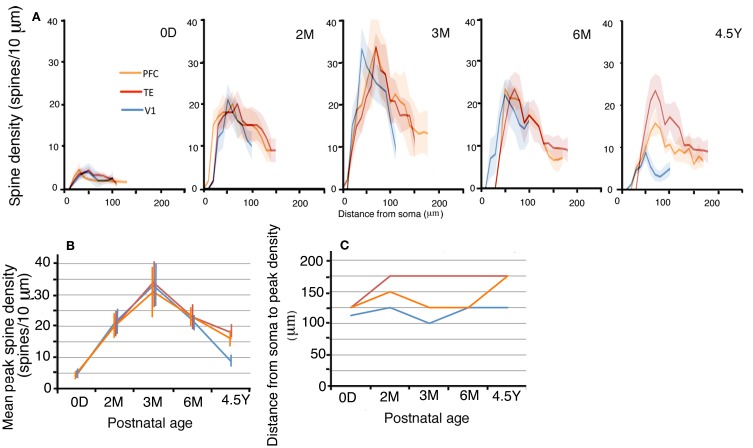
**(A)** Profiles of the spine densities of the basal dendrites of layer-III pyramidal cells as a function of distance from soma. The shaded areas indicate the standard deviations. The peak density (spine number/10 μm) **(B)**, the distance from the soma to the position of peak density **(C)** of layer-III pyramidal cells in V1, TE, and PFC at 0D, 2M, 3M, 6M, and 4.5Y. Error bars indicate standard deviations.

The layer III pyramidal cells in the three cortical areas exhibited increases in their spine density on their basal dendrites from 0D to 3M with similar time courses and similar amounts (Figures 5A,B; photomicrographs, Figure 2). This increase was evident along the entire extent of the dendrites (Figure 5A). The proximal dendritic segment that was 10–20 μm from the soma was devoid of spines throughout development in all areas. The distance at which spine density peaked was generally shorter for V1 than for TE, which was in turn shorter than for PFC (Figures 5A,C). During a period from 6M to 4.5Y, there was a considerable decrease in spine density in all cortical areas (Figures 5A,B). The decrease in spine density was greater in V1 than in TE and PFC (Figures 5A,B). Repeated measures ANOVA revealed that the differences in the spine densities across ages were significantly different in each cortical area (*p* < 0.001; V1, *F*_4_ = 180.08; TE, *F*_4_ = 109.02; PFC, *F*_4_ = 23.73). Repeated measures ANOVA also revealed significant differences (*p* < 0.05) in the spine densities among the cortical areas for a given age (0D, *F*_2_ = 6.681; 2M, *F*_2_ = 103.22; 3M, *F*_2_ = 6.627; 6M, *F*_2_ = 24.53; 4.5Y, *F*_4_ = 185.91).

**Figure 6:**
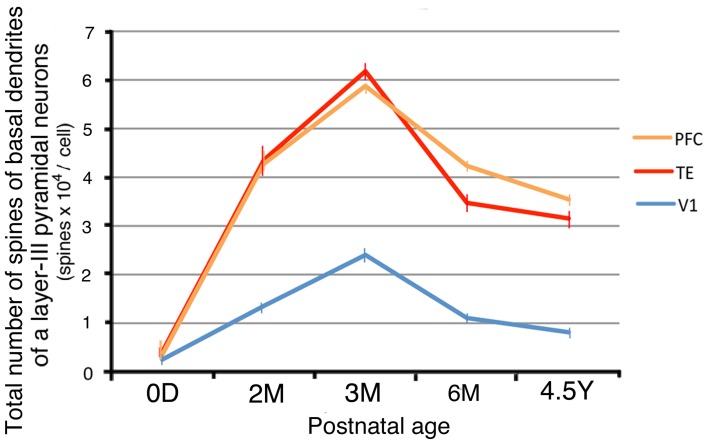
**The total number of spines in the basal dendritic trees of average layer-III pyramidal cells in V1, TE, and PFC at 0D, 2M, 3M, 6M, and 4.5Y.** Error bars indicate standard errors.

By combining the data from the Sholl analyses with those of the spine densities, we were able to calculate estimates for the total number of dendritic spines in the basal dendritic trees of the average pyramidal cells in each area for the different age groups (Elston et al., 2001). These calculations revealed dramatic differences in spinogenesis and pruning in the basal dendritic trees of layer III pyramidal cells across the three areas (Figure 6). Layer-III pyramidal cells in V1 had approximately 180 spines in their basal dendritic trees at 0D; this number increased to a maximum of 2300 at 3M and subsequently decreased to 950 at 4.5Y. Neurons in TE had 360 spines at 0D; this number increased to 6100 spines at 3M before decreasing to 3100 spines at 4.5Y. Neurons in PFC had 310 spines at 0D; this number increased to 5900 spines at 3M and then decreased to 3500 spines at 4.5Y. These results indicate that in all three areas, spine formation was greater than spine loss throughout development, resulting in a net increase in spine number in adults compared to newborns. However, the total number of spines in the basal dendrites and the net increase in V1 was markedly smaller than those in dendrites in TE and PFC (Figure 6).

### Consistency of the results across animals

All the above analyses were based on samples from one animal per age group except for 2M group in which data from three animals were pooled (Table 2). One-Way ANOVAs showed that all the parameters analyzed were not different between the three 2M animals [basal dendritic field area, *p* = 0.08 (*F*_2_ = 2.93); total basal dendrite length, *p* = 0.09 (*F*_2_ = 2.88); peak spine density, *p* = 0.30 (*F*_2_ = 1.28); estimated total spine dendrites, *p* = 0.14 (*F*_2_ = 2.88)].

## Discussion

We studied the postnatal development of the basal dendritic trees and their spines of layer-III pyramidal cells in V1, TE, and PFC. Neurons in TE and PFC had larger and more complex dendritic arbors than those in V1 at birth. The basal dendrites in TE and PFC then exhibited a marked increase in the tangential extent of their projection during the first 2 postnatal months, whereas those in V1 grew gradually until adulthood (Figure 3). The dendritic field was 2–3 times larger in TE and PFC than in V1 throughout all ages. Basal dendrites became more branched with age in all three areas, and they possessed more branches in TE and PFC than in V1 throughout development (Figure 4). The density of spines on a dendrite increased up to 3M and then decreased toward adulthood (Figure 5). This temporal profile was shared by all three areas, although the decline after 3M was more pronounced in V1 than in TE and PFC. The estimated total number of spines on basal dendrites reached a peak at 3M in all three areas (Figure 6). The spine number was higher in TE and in PFC than in V1 throughout all ages. The differences in pyramidal cell phenotype across V1, TE, and PFC in the adult previously reported (Elston et al., 1996, 2001; Elston, 2001) and confirmed in the present study thus derive both from the differences at birth and from the differential developmental processes.

### Comparison with macaque

The developmental profiles of layer-III pyramidal cells in the same three areas have recently been examined in the macaque monkey (*Macaca fascicularis*) (Elston et al., 2009, 2010a). These studies employed exactly the same method as that used in the present study, allowing us to make a direct comparison between the two species.

The developmental changes of the dendritic extent exhibited notable differences between the two species. In the macaque V1, the dendritic field was largest at birth and gradually decreased until adulthood, suggesting that the pruning of dendrites occurred (Elston et al., 2009). In contrast, the dendritic field of the neurons in the marmoset V1 gradually increased during development up until adulthood (Figure 3A). The dendritic growth pattern in PFC and TE also differed between the two species; in the macaque, the dendritic field gradually increased up until adulthood (Elston et al., 2009), whereas in the marmoset, an increase in the dendritic field occurred at or before 2 months and then remained unchanged (Figure 3A). Regarding the complexity of the dendritic branching that was assessed by Sholl analyses, TE and PFC neurons were more branched than V1 neurons in the marmoset (Figure 4) and in the macaque (Elston et al., 1996, 2009; Elston and Rosa, 1998; Elston, 2003), although no clear developmental trend was observed in the three areas of both species.

In contrast, the general developmental profile of spine density was strikingly similar between the marmoset and the macaque (Figure 5; for macaque data, see Figure 3 in Elston et al., 2009). In both species, V1, TE, and PFC showed similar density values at birth (approximately 5 spines/10-μm dendritic segment in the marmoset and 10 spines/10-μm dendritic segment in the macaque), and the spine densities concurrently increased and reached the maximum 3 months after birth. It was notable that the maximum spine densities exhibited similar values (30–35 spines/10-μm dendritic segment) between the two species and across diverse cortical areas, including V1 (present study; Elston et al., 2009), prestriate areas V2 and V4 (Elston et al., 2010a), inferior temporal subareas TEO (Elston et al., 2010a), TEpd and TEav (Elston et al., 2011), the primary auditory cortex (Elston et al., 2010b), and area 12 in the PFC (present study; Elston et al., 2009). The density then decreased toward adult values with different degrees among V1, TE, and PFC. In both species, spine density in V1 decreased most drastically. While the spine density in V1 was higher in adults than in newborns in the marmoset, it was lower in adults than in newborns in the macaque.

The developmental time courses of the total spine numbers on basal dendrites of layer-III pyramidal cells were also similar between V1, TE, and PFC and between the marmoset and the macaque. The total spine number peaked around 3M (Figure 6). The decline in the total number of spines after the peak was more prominent for the period of 3M–6M than for the period of 6M–4.5Y. The total spine number at any given age was the largest in PFC, which was followed by TE and then by V1 both in the marmoset and the macaque (Figure 6; for macaque, Elston et al., 2009).

For a given age/area group, the total spine number was smaller in the marmoset than in the macaque. In V1, the total number of spines was approximately 200 in the marmoset vs. 1900 in the macaque at birth, 2400 in the marmoset vs. 3900 in the macaque at 3–3.5M, and 800 in the marmoset and 900 in the macaque at adulthood. In TE, the counts were 400 in the marmoset and 3000 in the macaque at birth; 6200 in the marmoset and 10,400 in the macaque at 3–3.5M; and 3200 in the marmoset and 6200 in the macaque at adulthood. In PFC, the counts were 400 in the marmoset and 5000 in the macaque at birth; 5900 in the marmoset and 15,900 in the macaque at 3–3.5M; and 3600 in the marmoset and 8500 in the macaque at adulthood. A notable difference between the two species was that the total spine number in V1 was larger at birth than at adulthood in the macaque (Elston et al., 2009, 2010a), whereas, in the marmoset, similar to that in TE and PFC, the total spine number was larger at adulthood than at birth (Figure 6).

The ratio of the total spine number in the macaque divided by that of the marmoset tended to decrease gradually from birth to adulthood in the three areas (V1: birth, 1015%, 3M, 162%, adult, 112%; TE: birth, 728%, 3M, 168%, adult, 197%; PFC: 2D/0D, 1408%, 3M, 270%, adult, 238%). This calculation was based on Figure 6 in the present study and data from Elston et al. (2009, 2010a). The total number of spines on basal dendrites at birth was much larger in the macaque than in the marmoset. The difference at birth resulted from the 5-fold difference in the dendritic fields and the 2-fold difference in the spine densities between the two species.

The total number of spines on the basal dendrites in the adult marmoset was consistent between this and previous studies [V1: this study, 950; Elston et al., 1996, V1, 900; TE: this study, TEr, 3100; Elston et al., 1996, 3000 in TEc, 4200 in TEa (Thus, TEr in our study was similar to TEc in Elston et al., 1996); PFC: this study, 3500; Elston et al., 2001, 3800]. Furthermore, the measured values were not different among the three 2M animals in the present study. These results warranted the validity of our sampling and analytical methods. Although our data were based mostly on one animal per one age/area, we believe that the results are reliable.

### Comparison with human

A Golgi study showed that layer-III pyramidal cells in area 9 of the human PFC exhibited a peak spine density on the basal dendrites at 3 years of age (Petanjek et al., 2011). The total and mean distances of the basal dendrites of PFC layer-III pyramidal cells increased until 2 years of age and remained unchanged until adulthood (Petanjek et al., 2008). These results indicated that the age of the peak total spine number was approximately 2–3 years of age. The spines of the basal dendrites of pyramidal cells in human PFC undergo pruning over an extremely long period that spans 3 years of age to the third decade of life (Petanjek et al., 2011).

For humans, electron microscopy (EM) has been frequently used to examine synaptogenesis and pruning (Rakic et al., 1986; Huttenlocher, 1990; Zecevic and Rakic, 1991; Bourgeois and Rakic, 1993; Bourgeois et al., 1994; Huttenlocher and Dabholkar, 1997). Most excitatory inputs terminate on spines, and only a few excitatory synapses terminate on dendritic trunks (Missler et al., 1993). Thus, the number of spines is, in general, closely correlated to the number of excitatory synapses. Most EM studies have described synaptic density within a certain volume of neuropil as a measure. This measure is affected by the volume of other components in the neural tissue (axons, dendrites, and glial processes) and the total volume (including cell bodies and blood vessels) and the whole cortical area size (e.g., Rakic et al., 1986). In order to compare our data with data derived from EM studies, we need to know the quasi-total synapse number/one neuron and the whole volume of a cortical target area (at least the thickness of the cortex), the total number of neurons, and if possible, spine density on dendrites (Huttenlocher, 1990; Bourgeois and Rakic, 1993). Such combined data are available only in human V1 (Huttenlocher, 1990; Bourgeois and Rakic, 1993). With these parameters, Huttenlocher calculated the number of synapses per neuron separately for the different layers in V1. The number of synapses per layer III pyramidal cell rapidly increases from 4000 at birth to 18,000 at 8M (450% increase) and then decreases to 12,000 (62% decrease from 8M) at 18M. After 18M, the synapse number per layer III neuron is almost unchanged until 30 years of age (the eldest age examined in Huttenlocher, 1990). That is, layer-III pyramidal cells in human V1, in contrast to human PFC (Petanjek et al., 2011), do not exhibit gradual pruning from mid-childhood to adulthood. However, V1 in macaque monkeys show the opposite tendency: the total spine number is smaller in adults than in neonates (Elston et al., 2009). A previous study (Huttenlocher, 1990) reported the spine density on the basal dendrites of layer III pyramidal cells. The age of the peak density of spines on a dendrite is again 8M, and before and after 8M, there is a rapid increase and a decrease of density on dendrites, respectively. These features of the time courses of spine and/or synapse development is similar between the marmoset and human V1, except for the magnitude of synapse or spine number and the time points of the peak number of synapses or spines per neuron (in marmosets, at the peak time of 3M, the total spine number on basal dendrites of layer-III pyramidal cells was 2200 spines per neuron; in humans, at the peak time of 8M, there is 20,000 total synapses). This Golgi study (Huttenlocher, 1990) showed that the peak age of spine density on basal dendrites in V1 in human is later and the spine density is greater than that in the marmoset (in humans, at the peak time of 8M, the density is 12 synapses/μm; in marmosets, at the peak time of 3M, there was 0.35 spines/μm).

## Conclusion

Areas V1, TE, and PFC in the marmoset all exhibited an overshoot type of spine development with a peak at 3M. The dendritic field area and the total number of spines differed across ages and across areas. These results provide a starting point for studies of the molecular mechanisms of spinogenesis and pruning in the primate cerebral cortex. Comparisons among the transcriptomes of different time epochs of spine development in selected cortical areas are a strategic beginning for this endeavor. After selecting candidate genes, we can manipulate these genes with viral vector infection techniques or use the transgenic marmoset to assess their function in spine development.

## Conflict of interest statement

The authors declare that the research was conducted in the absence of any commercial or financial relationships that could be construed as a potential conflict of interest.

## References

[B1] Agustín-Pavón C. Braesicke K. Shiba Y. Santangelo A. M. Mikheenko Y. Cockroft G. (2012). Lesions of ventrolateral prefrontal or anterior orbitofrontal cortex in primates heighten negative emotion. Biol. Psychiatry 72, 266–272 10.1016/j.biopsych.2012.03.007 22502990

[B2] Bourgeois J. P. Goldman-Rakic P. S. Rakic P. (1994). Synaptogenesis in the prefrontal cortex of rhesus monkeys. Cereb. Cortex 4, 78–96 10.1093/cercor/4.1.78 8180493

[B3] Bourgeois J. P. Rakic P. (1993). Changes of synaptic density in the primary visual cortex of the macaque monkey from fetal to adult stage. J. Neurosci. 13, 2801–2820 8331373 10.1523/JNEUROSCI.13-07-02801.1993 PMC6576672

[B4] Broadbelt K. Byne W. Jones L. B. (2002). Evidence for a decrease in basilar dendrites of pyramidal cells in schizophrenic medial prefrontal cortex. Schizophr. Res. 58, 75–81 12363393 10.1016/s0920-9964(02)00201-3

[B5] Brodmann K. (1909). Vergleichende Lokalisationslehre der Großhirnrinde. Leipzig: Johann Ambrosius Barth

[B6] Burman K. J. Rosa M. G. (2009). Architectural subdivisions of medial and orbital frontal cortices in the marmoset monkey (*Callithrix jacchus*). J. Comp. Neurol. 514, 11–29 10.1002/cne.21976 19260047

[B7] Chandolia R. K. Luetjens C. M. Wistuba J. Yeung C. H. Nieschlag E. Simoni M. (2006). Changes in endocrine profile and reproductive organs during puberty in the male marmoset monkey (*Callithrix jacchus*). Reproduction 132, 355–363 10.1530/rep.1.01186 16885543

[B8] Eayrs J. T. Goodhead B. (1959). Postnatal development of the cerebral cortex in the rat. J. Anat. 93, 385–402 13819134 PMC1244533

[B9] Eliades S. J. Wang X. (2008). Neural substrates of vocalization feedback monitoring in primate auditory cortex. Nature 453, 1102–1106 10.1038/nature06910 18454135

[B10] Elston G. N. (2001). Interlaminar differences in the pyramidal cell phenotype in cortical areas 7m and STP (the superior temporal polysensory area) of the macaque monkey. Exp. Brain Res. 138, 141–152 10.1007/s002210100705 11417455

[B11] Elston G. N. (2003). Cortex, cognition and the cell: new insights into the pyramidal neuron and prefrontal function. Cereb. Cortex 13, 1124–1138 10.1093/cercor/bhg093 14576205

[B12] Elston G. N. Benavides-Piccione R. DeFelipe J. (2001). The pyramidal cell in cognition: a comparative study in human and monkey. J. Neurosci. 21, RC163 11511694 10.1523/JNEUROSCI.21-17-j0002.2001 PMC6763111

[B13] Elston G. N. Oga T. Fujita I. (2009). Spinogenesis and pruning scales across functional hierarchies. J. Neurosci. 29, 3271–3275 10.1523/JNEUROSCI.5216-08.2009 19279264 PMC6666449

[B14] Elston G. N. Oga T. Okamoto T. Fujita I. (2010a). Spinogenesis and pruning from early visual onset to adulthood: an intracellular injection study of layer III pyramidal cells in the ventral visual cortical pathway of the macaque monkey. Cereb. Cortex 20, 1398–1408 10.1093/cercor/bhp203 19846470

[B15] Elston G. N. Okamoto T. Oga T. Dornan D. Fujita I. (2010b). Spinogenesis and pruning in the primary auditory cortex of the macaque monkey (*Macaca fascicularis*): an intracellular injection study of layer III pyramidal cells. Brain Res. 1316, 35–42 10.1016/j.brainres.2009.12.056 20043887

[B16] Elston G. N. Oga T. Okamoto T. Fujita I. (2011). Spinogenesis and pruning in the anterior ventral inferotemporal cortex of the macaque monkey: an intracellular injection study of layer III pyramidal cells. Front. Neuroanat. 5:42 10.3389/fnana.2011.00042 21811440 PMC3143722

[B17] Elston G. N. Pow D. V. Calford M. B. (1997). Neuronal composition and morphology in layer IV of two vibrissal barrel subfields of rat cortex. Cereb. Cortex 7, 422–431 10.1093/cercor/7.5.422 9261572

[B18] Elston G. N. Rosa M. G. (1998). Morphological variation of layer III pyramidal neurones in the occipitotemporal pathway of the macaque monkey visual cortex. Cereb. Cortex 8, 278–294 9617923 10.1093/cercor/8.3.278

[B19] Elston G. N. Rosa M. G. P. (1997). The occipitoparietal pathway of the macaque monkey: comparison of pyramidal cell morphology in layer III of functionally related cortical visual areas. Cereb. Cortex 7, 432–452 10.1093/cercor/7.5.432 9261573

[B20] Elston G. N. Rosa M. G. Calford M. B. (1996). Comparison of dendritic fields of layer III pyramidal neurons in striate and extrastriate visual areas of the marmoset: a Lucifer yellow intracellular injection. Cereb. Cortex 6, 807–813 8922337 10.1093/cercor/6.6.807

[B21] Fritsches K. A. Rosa M. G. (1996). Visuotopic organization of striate cortex in the marmoset monkey (*Callithrix jacchus*). J. Comp. Neurol. 372, 264–282 10.1002/(SICI)1096-9861(19960819)372:2<264::AID-CNE8>3.0.CO;2-1 8863130

[B22] Garey L. (2010). When cortical development goes wrong: schizophrenia as a neurodevelopmental disease of microcircuits. J. Anat. 217, 24–33 10.1111/j.1469-7580.2010.01231.x 20408906 PMC2992411

[B23] Glantz L. A. Lewis D. A. (2000). Decreased dendritic spine density on prefrontal. cortical pyramidal neurons in schizophrenia. Arch. Gen. Psychiatry 57, 65–73 10.1001/archpsyc.57.1.65 10632234

[B24] Hutsler J. J. Zhang H. (2010). Increased dendritic spine densities on cortical projection neurons in autism spectrum disorders. Brain Res. 1309, 83–94 10.1016/j.brainres.2009.09.120 19896929

[B25] Huttenlocher P. R. (1990). Morphometric study of human cerebral cortex development. Neuropsychologia 28, 517–527 10.1016/0028-3932(90)90031-I 2203993

[B26] Huttenlocher P. R. Dabholkar A. S. (1997). Regional differences in synaptogenesis in human cerebral cortex. J. Comp. Neurol. 387, 167–178 10.1002/(SICI)1096-9861(19971020)387:2<167::AID-CNE1>3.0.CO;2-Z 9336221

[B27] Kaas J. H. (1997). Theories of visual cortex organization in primates, in Cerebral Cortex. Extrastriate Cortex in Primates, Vol. 12, eds Rockland K. Kaas J. H. Peters A. (New York, NY: Plenum), 91–125

[B28] Kalus P. Müller T. J. Zuschratter W. Senitz D. (2000). The dendritic architecture of prefrontal pyramidal neurons in schizophrenic patients. Neuroreport 11, 3621–3625 11095531 10.1097/00001756-200011090-00044

[B29] Kaufmann W. E. Moser H. W. (2000). Dendritic anomalies in disorders associated with mental retardation. Cereb. Cortex 10, 981–991 10.1093/cercor/10.10.981 11007549

[B30] Levenga J. Willemsen R. (2012). Perturbation of dendritic protrusions in intellectual disability. Prog. Brain Res. 197, 153–168 10.1016/B978-0-444-54299-1.00008-X 22541292

[B31] McKinnell C. Saunders P. T. Fraser H. M. Kelnar C. J. Kivlin C. Morris K. D. (2001). Comparison of androgen receptor and estrogen receptor beta immunoexpression in the testes of the common marmoset (*Callithrix jacchus*) from birth to adulthood: low androgen receptor immunoexpression in Sertoli cells during the neonatal increase in testosterone concentrations. Reproduction 122, 419–429 10.1530/rep.0.1220419 11597306

[B32] Micheva K. D. Beaulieu C. (1996). Quantitative aspects of synaptogenesis in the rat barrel field cortex with special reference to GABA circuitry. J. Comp. Neurol. 373, 340–354 10.1002/(SICI)1096-9861(19960923)373:3<340::AID-CNE3>3.0.CO;2-2 8889932

[B33] Missler M. Wolff A. Merker H. J. Wolff J. R. (1993). Pre- and postnatal development of the primary visual cortex of the common marmoset. II., Formation, remodelling, and elimination of synapses as overlapping processes. J. Comp. Neurol. 333, 53–67 10.1002/cne.903330105 8340496

[B34] Paxinos G. Watson C. Petrides M. Rosa M. Tokuno H. (2011). The Marmoset Brain in Stereotaxic Coordinates. London: Academic Press

[B35] Penzes P. Cahill M. E. Jones K. A. VanLeeuwen J. E. Woolfrey K. M. (2011). Dendritic spine pathology in neuropsychiatric disorders. Nat. Neurosci. 14, 285–293 10.1038/nn.2741 21346746 PMC3530413

[B36] Petanjek Z. Judas M. Kostovi'c I. Uylings H. B. (2008). Lifespan alterations of basal dendritic trees of pyramidal neurons in the human prefrontal cortex: a layer-specific pattern. Cereb. Cortex 18, 915–929 10.1093/cercor/bhm124 17652464

[B37] Petanjek Z. Judaš M. Šimic G. Rasin M. R. Uylings H. B. Rakic P. (2011). Extraordinary neoteny of synaptic spines in the human prefrontal cortex. Proc. Natl. Acad. Sci. U.S.A. 108, 13281–13286 10.1073/pnas.1105108108 21788513 PMC3156171

[B38] Rakic P. Bourgeois J. P. Eckenhoff M. F. Zecevic N. Goldman-Rakic P. S. (1986). Concurrent overproduction of synapses in diverse regions of the primate cerebral cortex. Science 232, 232–235 10.1126/science.3952506 3952506

[B39] Rosa M. G. Palmer S. M. Gamberini M. Burman K. J. Yu H. H. Reser D. H. (2009). Connections of the dorsomedial visual area: pathways for early integration of dorsal and ventral streams in extrastriate cortex. J. Neurosci. 29, 4548–4563 10.1523/JNEUROSCI.0529-09.2009 19357280 PMC6665729

[B40] Sasaki E. Suemizu H. Shimada A. Hanazawa K. Oiwa R. Kamioka M. (2009). Generation of transgenic non-human primates with germline transmission. Nature 459, 523–527 10.1038/nature08090 19478777

[B41] Sholl D. A. (1955). The surface area of cortical neurons. J. Anat. 89, 571–572

[B42] Snowdon C. T. (1997). Affiliative processes and vocal development. Ann. N.Y. Acad. Sci. 807, 340–351 10.1111/j.1749-6632.1997.tb51931.x 9071362

[B43] Valverde F. (1967). Apical dendritic spines of the visual cortex and light deprivation in the mouse. Exp. Brain Res. 3, 337–352 6031165 10.1007/BF00237559

[B44] van Spronsen M. Hoogenraad C. C. (2010). Synapse pathology in psychiatric and neurologic disease. Curr. Neurol. Neurosci. Rep. 10, 207–214 10.1007/s11910-010-0104-8 20425036 PMC2857788

[B45] Zecevic N. Rakic P. (1991). Synaptogenesis in monkey somatosensory cortex. Cereb. Cortex 1, 510–515 10.1093/cercor/1.6.510 1822755

